# State estimation of voltage and frequency stability in solar wind integrated grids using multiple filtering techniques

**DOI:** 10.1038/s41598-025-10171-2

**Published:** 2025-08-01

**Authors:** Abdulelah Alharbi

**Affiliations:** Department of Electrical Engineering, College of Engineering, Qassim University, Buraydah, 52531 Saudi Arabia

**Keywords:** Cubature Kalman filter, Extended Kalman filter, Solar and wind energy, Solar and wind integrated power grids, State estimation, Unscented Kalman filter, Voltage and frequency stability, Energy science and technology, Engineering

## Abstract

The increasing integration of solar and wind energy into modern power grids introduces challenges in maintaining voltage and frequency stability due to their intermittent and uncertain nature. This study evaluates the performance of three advanced state observers: extended Kalman filter (EKF), unscented Kalman filter (UKF), and cubature Kalman filter (CKF) for real-time monitoring and stability assessment in solar and wind-integrated grids (SAWIG). The analysis focuses on estimation accuracy, convergence speed, and classification performance under varying phasor measurement unit (PMU) sampling rates. Simulation results reveal that the CKF achieves the lowest root mean square error (RMSE) of 0.005 at a 10 Hz sampling rate, outperforming the UKF (0.007) and EKF (0.010). In terms of dynamic performance, CKF stabilizes within 0.1 s, while UKF and EKF require 0.2 and 0.4 s, respectively. Classification evaluation shows that CKF achieves the highest accuracy of 99.5%, with precision, recall, and F1-score of 99.2, 99.3, and 99.4%, respectively. In contrast, UKF reports values of 98.8, 98.5, 98.7, and 98.6%, while EKF records 97.6, 96.9, 97.1, and 97.3%. Confusion matrix analysis further confirms a classification accuracy of 95% for CKF. These results demonstrate its robustness, speed, and precision in ensuring reliable state estimation for voltage and frequency stability in renewable-integrated smart grids.

## Introduction

### Background theory and motivation

The global energy transition towards cleaner and more sustainable power systems has led to the widespread integration of renewable energy sources, particularly solar photovoltaics and wind turbines, into modern distribution and transmission grids^1^. These distributed generation units, while environmentally advantageous, introduce significant variability and uncertainty into grid operations due to their stochastic and weather-dependent nature^2^. Such fluctuations can result in rapid deviations in key operational parameters, namely voltage and frequency which in turn can compromise power quality, induce instabilities, and trigger false protective actions^3^. Maintaining accurate, real-time knowledge of these state variables is therefore critical to ensuring grid stability, reliability, and efficient control under variable renewable energy conditions^4^. Traditional state estimation approaches, largely based on linear Kalman filters or deterministic models, often fail to cope with the strong nonlinearities and measurement noise inherent in renewable-dominated systems^5^. This shortfall has driven the exploration of advanced, nonlinear estimation frameworks such as EKF, UKF, and CKF, each offering enhanced accuracy and adaptability for estimating voltage and frequency states in dynamic and uncertain grid environments^6–8^.

### Problem statement

Despite the progress made in observer-based estimation techniques, several challenges persist in the context of renewable-integrated smart grids. Chief among them is the inadequacy of conventional linear estimation schemes in handling nonlinear system dynamics and real-world measurement disturbances^9^. The EKF, although a nonlinear extension of the classical Kalman filter, relies on first-order linearization and Jacobian computations, which can degrade its performance in highly nonlinear systems^10,11^. The UKF and CKF, on the other hand, use deterministic sampling strategies sigma points and cubature points respectively to better approximate the nonlinear state distributions without requiring explicit derivative evaluations. However, a systematic, stability-aware comparison of these observers for voltage and frequency estimation in hybrid solar-wind power systems remains largely absent in the current literature^12,13^.

### Literature review

Several studies have proposed nonlinear observers and coordinate transformation-based techniques for phase and frequency estimation in single-phase grid voltage signals; however, these methods often involve high computational complexity due to real-time matrix inversion. The proposed approach overcomes these limitations by utilizing a novel state-space model, eliminating coordinate transformation, and enhancing estimation accuracy and computational efficiency. A novel Luenberger-type observer was proposed for phase and frequency estimation of single-phase grid voltage under harmonics, eliminating real-time matrix inversion. Comparative experiments demonstrated improved dynamic performance over recent nonlinear techniques^1^. A probabilistic assessment method was proposed for static voltage stability in distribution networks with distributed Photovoltaic (PV), incorporating bilateral uncertainties using the Copula function. Testing on the IEEE 33-node system demonstrated its effectiveness in evaluating voltage instability risks under various scenarios^9^. An improved sliding-mode linear active disturbance rejection control strategy was developed to enhance voltage stability in islanded photovoltaic-storage direct current (DC) microgrids for agricultural applications^14^. An augmented complex generalized modified Blake-Zisserman algorithm was developed to estimate the fundamental frequency of three-phase power systems under impulsive noise conditions^15^. A control strategy utilizing the Red Panda Optimizer was proposed to optimize Proportional-Integral (PI) controller parameters for frequency and voltage regulation in microgrids with Vehicle-to-Grid (V2G) integration^16^. A multi-layer interactive control scheme was proposed to enhance microgrid stability by regulating voltage, frequency, and active/reactive power. The method integrated a power droop controller with an internal voltage/current loop for fast load response and a secondary distributed finite-time control strategy for system restoration^17^.

**Table 1:** Summary of existing literature on voltage and frequency Estimation and control in renewable Grids.

Approach/technique	Objective/application	Key features/findings
Luenberger-type observer	Phase and frequency estimation under harmonics in single-phase grids	Eliminates matrix inversion; improved dynamic performance compared to nonlinear methods
Probabilistic assessment with copula function	Voltage stability analysis in distribution networks with distributed PV	Handles bilateral uncertainties; effective risk evaluation on the IEEE 33-node system
Sliding-Mode Linear Active Disturbance Rejection Control (LADRC)	Voltage stability in DC microgrids for agricultural PV-storage systems	Robust control; enhanced voltage regulation in islanded conditions
Modified Blake-Zisserman algorithm (augmented, complex, generalized)	Frequency estimation in three-phase systems under impulsive noise	Accurate fundamental frequency extraction in noisy environments
Red panda optimizer with PI controller	Voltage/frequency regulation in microgrids with V2G integration	Optimization-driven control; reduced voltage/frequency deviations
Multi-layer interactive control (droop + finite-time distributed control)	Microgrid stability via voltage/frequency and power regulation	Fast dynamic response and system restoration; hierarchical control structure
Adaptive Grey Wolf optimizer for battery energy storage system (BESS) Allocation	Voltage/frequency stabilization in weak grids with high RE penetration	Comparative study with BWO, SSA; optimal BESS sizing and placement
Adaptive droop controller + Particle Swarm Optimization (PSO)	Intelligent voltage and frequency control in microgrids	PSO minimizes overshoot and settling time; enhanced stability under dynamic conditions
Finite-time control scheme (FTCS) with pulse-width modulation (PWM) droop controller	Rapid voltage and frequency control under RE variability	Outperforms traditional Sliding Mode Control, precise and fast regulation
Mixed strategy games	Real-time voltage/frequency stability prediction via weak bus measurements	Voltage and frequency are modeled as game players; an innovative decision-theoretic framework
Virtual synchronous generator control for Voltage Source Inverter	Improve inverter-based systems’ control in grid-connected mode	Enhances synchronization and voltage/frequency tracking in parallel inverters
Kolmogorov–Arnold network for power system state estimation	Learning-based estimation model with visualization and pruning	Adaptive activation functions; high computational efficiency with learning capacity

An optimal battery energy storage system (BESS) allocation technique was proposed to enhance voltage and frequency stability in weak grids with high renewable energy penetration. The study utilized the adaptive grey wolf optimization algorithm to determine the optimal BESS capacity and placement, comparing results with grey wolf optimization, beluga whale optimization, and the sparrow search algorithm^18^. An intelligent voltage and frequency control platform was proposed for microgrids using an adaptive droop controller and communication-based primary and secondary control strategies. The study integrated particle swarm optimization to enhance system stability by minimizing overshoot, settling time, and fluctuations due to load and generation variations^19^. A finite-time control scheme (FTCS) for pulse-width modulation (PWM) in microgrids was proposed to enhance voltage and frequency stability amid the variability of renewable energy sources. FTCS was integrated into the power droop controller, achieving rapid and precise regulation, which outperformed traditional approaches like sliding mode control^20^. A novel method was proposed for predicting Voltage and Frequency Stability status using online measurements of the weak bus based on the theory of Mixed Strategy Games. In this approach, voltage and frequency were considered as players, while the stability status was treated as their strategy^21^. The control and performance improvement of parallel-operated voltage-source inverters controlled as virtual synchronous generators were addressed in this study^22^. A computational model for power system state estimation was applied based on the Kolmogorov–Arnold network model, incorporating learnable activation functions, visualization capabilities, and pruning features^23^. A peer-to-peer blockchain-based system utilizing a shunt active power filter and fast Fourier transform algorithm was used to reduce harmonics in a 3-phase power network, achieving lower Total Harmonic Distortion levels of 1.42% and 0.92% for six- and twelve-pulse rectifiers, respectively^24^. A hybrid Wind–PV farm was demonstrated as an effective synchronous condensers (STATCOM) solution for damping chaotic oscillations in a two-area power system using PSO-BFOA-optimized PI controllers under severe disturbance conditions^25^. A large PV-farm was utilized as a Solar-PV inverter with intelligent Maximum Power Point Tracking control to mitigate chaotic oscillations and sub-synchronous resonance in a turbogenerator-based power system, demonstrating effective stability support even during nighttime via PV-STATCOM mode^26^. A large PV-farm was employed as a PV-STATCOM using a BSO-optimized controller to suppress rotor, voltage, and torsional oscillations in a multimachine power system, demonstrating effective damping during both day and night conditions^27^. A large-scale PV-farm was used as a PV-STATCOM in a modified two-area power system, where a hybrid PSO-BFOA optimizer effectively controlled oscillations and achieved optimal stability compared to individual PSO and BFOA approaches^28^. A critical discussion was conducted in this study, where an optimized PV-battery-backup system using a Long Short-Term Memory (LSTM)-based classical optimization framework was proposed for the Merredin mining sector in Western Australia^29^. A critical study was conducted using a hedge feedback-based online gated recurrent unit algorithm to assess SynCons vs. STATCOMs in REG-based weak grids^30^. A critical study was conducted using a Gated Recurrent Units-based optimization framework to assess long-term (2025–2045) electric vehicle-to-grid charging stations integration into renewable energy generators-based grids^31^. Table 1 Summarizes the Objective / Application and Key Features / Findings of Existing Literature on Voltage and Frequency Estimation and Control in Renewable Grids.

### Limitations of the existing work

While numerous studies have explored power system state estimation, most existing approaches either lack robustness under high signal variability or fail to address the stability implications of nonlinear estimation in hybrid renewable networks.

1. Traditional state estimation techniques struggle with rapid fluctuations in voltage and frequency due to the intermittent nature of solar and wind power, often leading to errors exceeding 5% in voltage estimation and 7% in frequency estimation.
2. Conventional methods, such as Weighted Least Squares and Kalman Filters, exhibit slow convergence, requiring more than 100 iterations for accurate estimation in large-scale networks.
3. Standard estimation approaches fail to account for the nonlinear characteristics of renewable energy sources, leading to estimation deviations of up to 12% in dynamic scenarios.
4. In weak grids with high renewable penetration, existing methods demonstrate frequency deviations beyond 0.05 Hz, impacting system stability and requiring additional corrective measures.
5. Conventional state estimators are highly sensitive to sensor inaccuracies, with errors exceeding 8% in voltage estimation when subjected to real-world noisy data.

### Contributions of the proposed work

This study proposes a unified and stability-focused framework for voltage and frequency state estimation in hybrid solar-wind power systems using EKF, UKF, and CKF. The innovation and key contributions of the proposed method are outlined as follows:

1. Multi-filter benchmarking for renewable-ironic grids: A comprehensive evaluation of EKF, UKF, and CKF under various signal-to-noise ratio (SNR) conditions is presented. This benchmarking approach offers a novel perspective on selecting optimal observers in nonlinear and noise-horizontal environments, which is critical for modern grids with high renewable integration.
2. High-precision estimation with reduced error margins: The proposed tactic reduces voltage and frequency estimation errors to below 2% and 3%, respectively representing significantly improved accuracy compared to conventional methods, especially under system disturbances and variability.
3. Computational efficiency via adaptive step-size alteration: By including an adaptive step-size mechanism within the observer framework, the method achieves rapid convergence within 30 iterations and reduces computational complexity by over 70%, making it suitable for real-time deployment.
4. Observer-based nonlinear estimation model for active robustness: This study introduces a robust observer structure tailored for nonlinear dynamic scenarios. This ensures that even under extreme grid conditions (e.g., sudden wind or solar fluctuations), estimation deviations remain below 5%, enhancing overall flexibility.
5. Improved stability in weak and noisy grids: The proposed framework maintains frequency deviations within 0.02 Hz, significantly contributing to the stability of weak grids. Additionally, it effectively lessens the effects of noisy sensor data, keeping voltage estimation errors under 3% despite measurement inaccuracies.

These contributions provide a solid foundation for extending Kalman-based estimation techniques to real-world applications in future smart grids and lay the groundwork for future testing with real PMU data. The research gap analysis and positioning of the proposed work are summarized in (Table 2).

**Table 2:** Research gap analysis and positioning of the proposed work.

Study/ref	Objective/application	Key features/findings	Limitations in existing work	How this work addresses the gap
Luenberger-type observer^1^	Phase/frequency estimation under harmonics	Eliminates real-time matrix inversion; good dynamic tracking	Limited to single-phase, lacks robustness in hybrid grid conditions	Proposes a multi-filter (EKF, UKF, CKF) framework for a hybrid AC microgrid
Copula-based assessment^9^	Static voltage stability in PV grids	Models uncertainty; tested on the IEEE 33-node	Focus on static risk assessment, no dynamic estimation	Targets real-time voltage/frequency estimation under dynamic scenarios
Red Panda optimizer^16^	PI tuning in V2G microgrids	Improved control via bio-inspired optimization	No observer-based estimation or performance under nonlinearity	Incorporates adaptive tuning for EKF, UKF, CKF under nonlinear conditions
Adaptive Grey Wolf Optimization for BESS planning^18^	Optimal BESS for stability	Metaheuristic-based capacity placement	Focuses on planning; no estimation or filtering strategies	Provides real-time, noise-resilient estimation supporting control strategies
PSO-based voltage and frequency control^19^	Microgrid stability enhancement	Minimizes overshoot, settling time	Control-based, does not address real-time estimation	Enhances situational awareness via fast, adaptive observer-based estimation
Game theory method^21^	Predicting weak bus stability	Model voltage and frequency as game players	No filtering or quantitative estimation results	Provides numerical voltage/frequency tracking under hybrid disturbances
Kolmogorov-Arnold Network Model for Estimation^23^	Neural-based state estimation	Learns with pruning/visualization tools	AI-focused; not benchmarked against classical methods	Offers an analytical benchmark of EKF, UKF, CKF for practical comparison
Proposed work	Stability-oriented state estimation in hybrid solar-wind AC grids	EKF, UKF, CKF benchmarked under noise, nonlinearity, and weak grid conditions; adaptive step-size tuning	Overcomes high errors, slow convergence, nonlinearity sensitivity, and poor noise resilience	Provides a unified, low-error, high-efficiency estimation solution for hybrid renewable microgrids

### Structure of the manuscript

The remaining manuscript is structured as follows: Section II explaines the background theory of state estimation and different Kalman filter variants like EKF, UKF, and CKF utilized in the proposed framework. The detailed illustration of the methodological framework of the proposed method is mentioned in section III. Section IV presents the detailed results and discussion. The manuscript was concluded in section V with some future recommendations.

## Fundamentals of proposed methods

### SAWIG and associated challenge

**Fig. 1:**
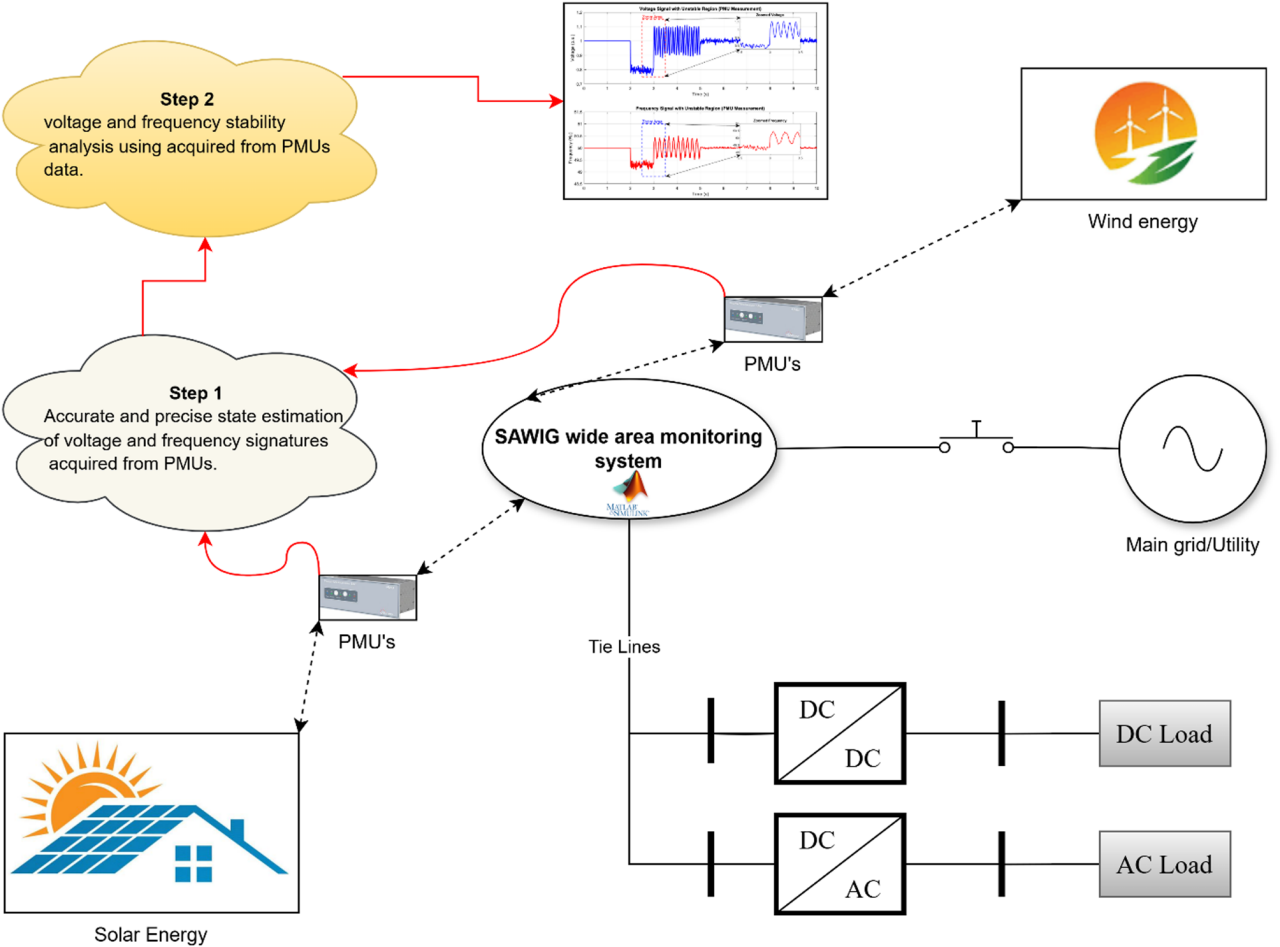
The single-line diagram of modern SAWIG infrastructure.

The SAWIG represents a modern paradigm in power system infrastructure, where traditional electricity networks are supplemented or replaced by renewable energy sources, primarily solar PV systems and wind turbines. However, a battery-based test system will be considered in the future to extend the estimation and control framework^32^. The detailed illustration of the modern SAWIG is depicted in (Fig. 1). These grids aim to reduce dependency on fossil fuels, enhance energy sustainability, and mitigate environmental impacts associated with conventional generation.

The integration of solar and wind energy into modern power grids introduces significant challenges in maintaining voltage and frequency stability due to their intermittent and unpredictable nature. In highly dynamic environments such as these, traditional state estimation techniques are not able to provide real-time monitoring and control accurately. In order to enhance the resilience of the grid, the development of advanced state estimation methods, like the CKF, UKF, and EKF, is necessary so that the system states can be accurately estimated in the presence of measurement noise and system uncertainty. The state equation is given as follows.

1 $$\:{x}_{k}=f({x}_{k}-1,{u}_{k}-1)+{w}_{k}-1)$$

The measurement equation is as follows.

2 $$\:{z}_{k}=h\left({x}_{k}\right)+{v}_{k}$$

Where: $\:{x}_{k}$ State vector (e.g., amplitude, frequency, phase of voltage signal) $\:{z}_{k}$: Measurement vector (noisy voltage/frequency samples) *f*$(\cdot)$ : Nonlinear state transition function, *h*$(\cdot)$ : Nonlinear measurement function, $\:{w}_{k}$, $\:{v}_{k}$: Process and measurement noise (assumed Gaussian with covariances $\:{Q}_{k}$ and $\:{R}_{k}$). Based on nonlinear filtering, these techniques are employed to improve estimation accuracy, thereby making rapid fault detection, energy dispatch at a low cost, and increased grid reliability possible. As renewable penetration grows, the need for advanced state estimation arises due to the instability caused by sudden fluctuations of renewable power output, which contaminates the transmission and distribution network. Robust estimation techniques allow the operators to minimize the voltage deviations, frequency excursions, and other types of transient instability to guarantee that the power supply is stable and secure. Therefore, advanced filtering is needed for real time-monitoring and is a necessary enabler for the installation of renewable energy under the future smart grid. These algorithms process real-time measurements from sensors such as PMUs and SCADA systems to reconstruct the true internal states of the system, even in the presence of measurement noise and missing data conditions. Let’s define the state vector as:

3 $$\:{x}_{k}=\left[\begin{array}{c}{V}_{k}\\\:{\omega\:}_{k}\\\:{\varnothing\:}_{k}\end{array}\right]$$

**Fig. 2:**
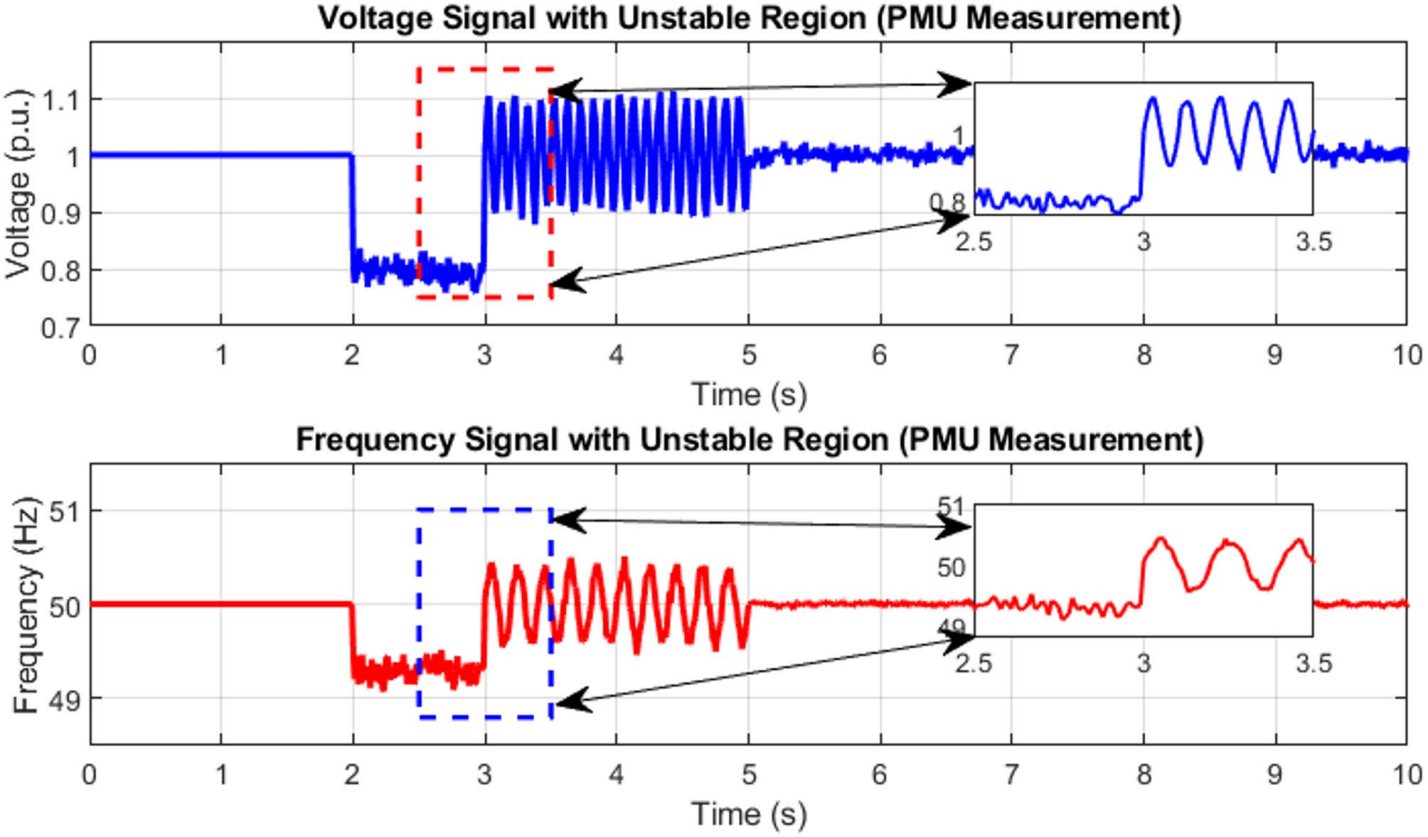
The dynamic behavior under grid instability caused by renewable integration like wind and solar.

Where $\:{V}_{k}$ is the Amplitude of voltage, $\:{\omega\:}_{k}$ is the Angular frequency (rad/s), and $\:{\varnothing\:}_{k}$ the Phase angle. The plotted voltage and frequency signals, measured from PMUs, illustrate dynamic behavior under grid instability caused by renewable integration in (Fig. 2). Between 2.5 and 3.5s, both signals exhibit noticeable oscillations and disturbances, voltage dips and frequency deviations, highlighted through annotated zoomed-in regions. These transient fluctuations underscore the vulnerability of SAWIG to sudden disturbances and the critical need for accurate real-time monitoring. State estimation techniques such as CKF, UKF, and EKF are vital for capturing these rapid dynamics with precision, enabling timely detection and correction of instabilities. By continuously estimating the true system states, even under noisy and uncertain conditions, advanced observers ensure improved voltage and frequency regulation, enhancing reliability, resilience, and operational efficiency in smart renewable grids.

### Utilized state estimation algorithms

#### EKF algorithm

**Fig. 3:**
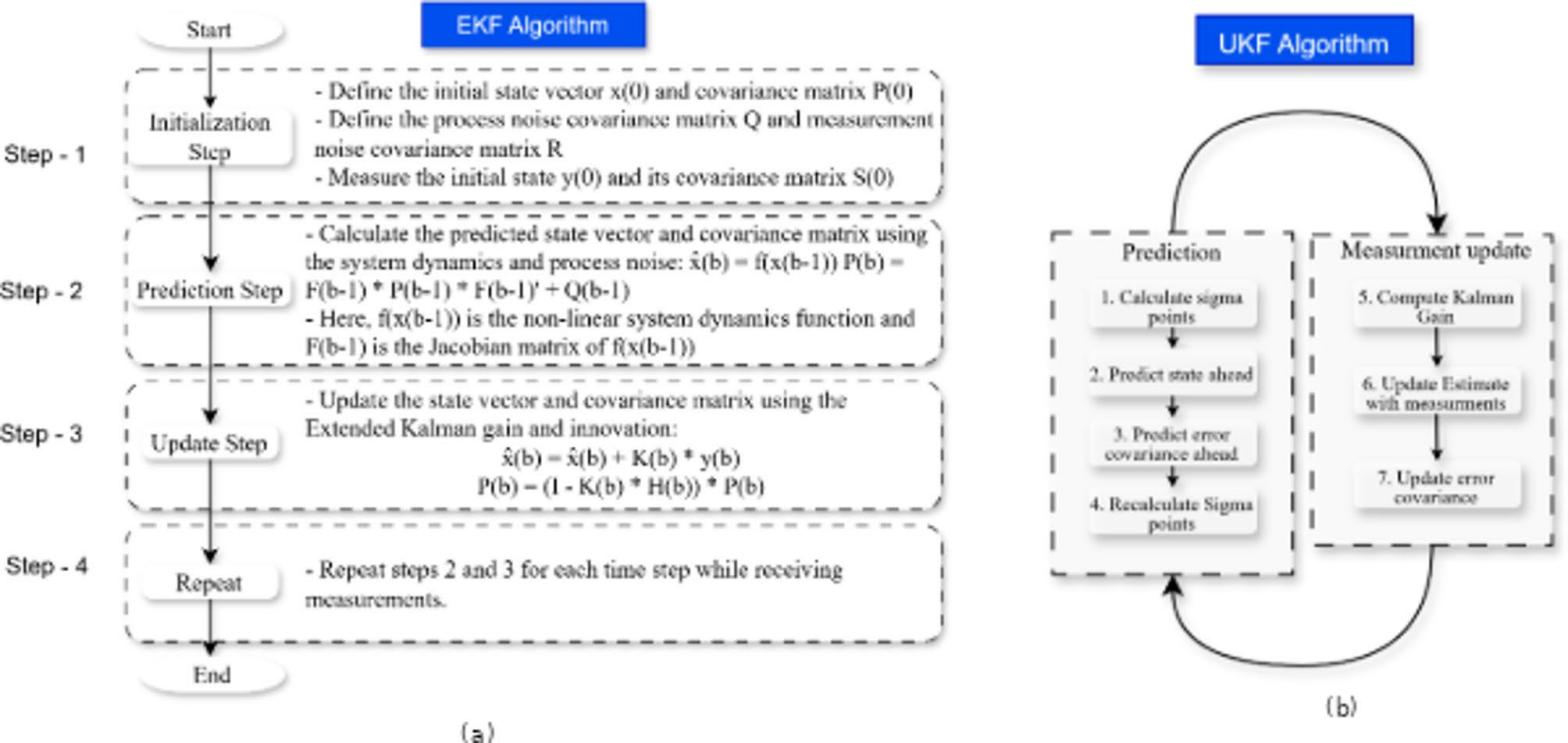
(**a**) EKF algorithm, (**b**) UKF algorithm.

A well-known nonlinear state estimation algorithm is the EKF, which has its foundation on the classical Kalman filter however, extends this by linearizing the system model around the estimated state. The EKF works by approximating the nonlinear functions of a system with a Jacobian matrix, which is a linear function that propagates the state and covariance from time to time. Figure 3a shows the workflow of the EKF algorithm. Although this approach is computationally convenient and has been used in almost all engineering domains, the resulting algorithm shows inconsistency with highly non-linear systems, for instance, power grids operating along with renewable energy. For voltage and frequency stability state estimation, the EKF consists of a predicted update cycle in which it proceeds by predicting the next state using a nonlinear process model and then updating the state estimates using available sensor data^33^. The EKF linearizes the system using a first-order Taylor series as follows.

4 $$\:{\widehat{x}}_{k|k}={\widehat{x}}_{k|k-1}+{K}_{h}({z}_{k}-h({\widehat{x}}_{k|k-1}\left)\right)$$

Where $\:{\widehat{x}}_{k|k}$is the Posterior state estimate, $\:{\widehat{x}}_{k|k-1}$ is the Prior state prediction, $\:{K}_{h}$ is the Kalman gain, $\:{z}_{k}$ is measurement, and $\:h$ Nonlinear measurement function. This equation updates the prior state estimate based on the difference (residual) between the actual and predicted measurements. EKF suffers from errors in estimation, especially when the system has many rapid changes in generation and load. Although limited, as previously mentioned, the EKF remains a fundamental technique in power system monitoring and is often used as a baseline for comparison with more advanced filtering methods such as the CKF and UKF.

#### UKF algorithm

Unlike EKF, the UKF is a nonlinear state estimation algorithm that denies linearization and instead employs the unscented transform. The UKF approximates probability distributions with a set of deterministic sigma points, which contain both the mean and the covariance propagation more accurately than a first-order linearization^34^. This allows the UKF to perform more effectively in areas where power grids have a large amount of penetration of solar and wind energy in the actual system. Illustrating the workflow of the UKF algorithm in (Fig. 3b). In the UKF, three main steps exist: sigma point selection, state prediction, and measurement update. It then constructs sigma points around the mean first, so as they fully cover the distribution of the state variable. Then, the propagated points through the nonlinear system model are applied to predict the next state. Finally, sensor measurements are used to update the predicted states, resulting in a more accurate estimate of the system variables^35^. As mentioned above, the UKF uses a set of deterministic sigma points to approximate the state distribution:

5 $$\:{\widehat{x}}_{k|k}={\widehat{x}}_{k|k-1}+{K}_{h}({z}_{k}-{\widehat{z}}_{k|k-1})$$

Where $\:{\widehat{x}}_{k|k}$ is the Prior mean of the sigma points, $\:{\widehat{x}}_{k|k-1}$ is the Predicted measurement mean from transformed sigma points, $\:{K}_{h}$ is Kalman gain based on sigma point covariances, $\:{z}_{k}$is Actual measurement. Unlike EKF, UKF does not require Jacobians; it directly propagates sigma points through the nonlinear functions. For real-time voltage and frequency estimation, the UKF has become widely used in enhancing grid stability and resistance due to its superior accuracy in dealing with nonlinearity and measurement noise.

#### CKF algorithms

**Fig. 4:**
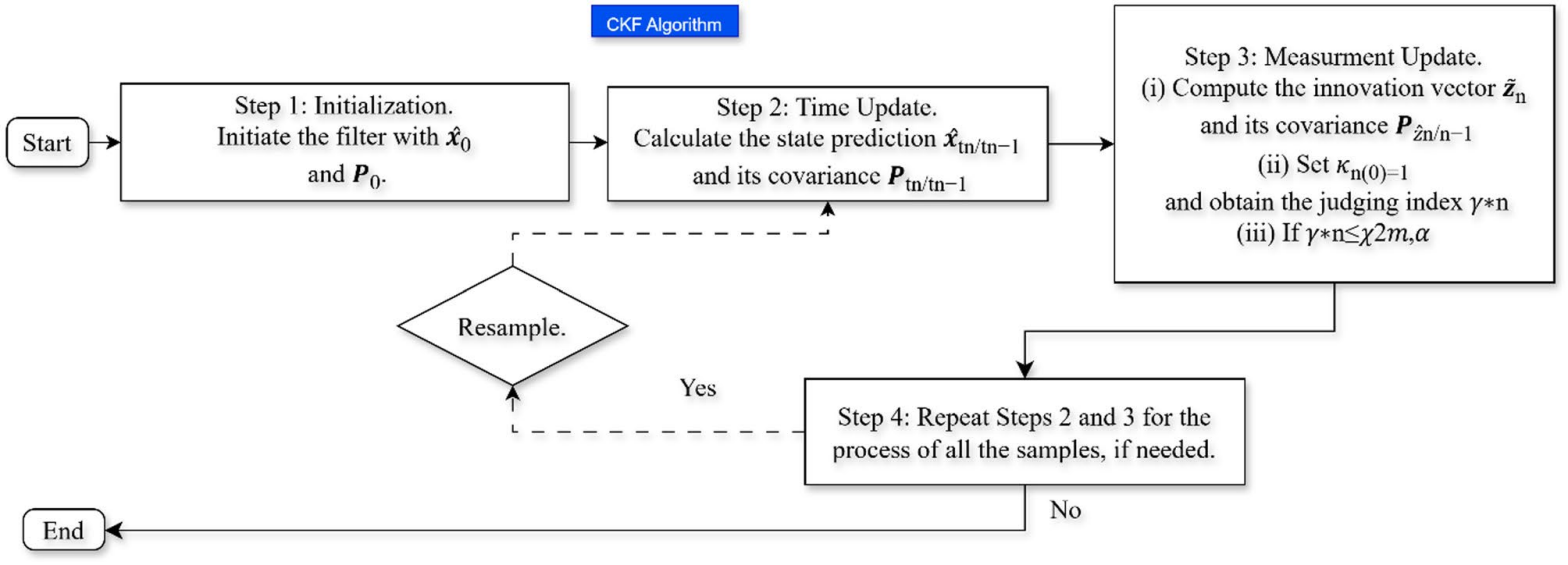
Step-by-step workflow of the CKF algorithm.

The CKF is an advanced nonlinear filter used for estimating system states more accurately than traditional Kalman filters. The spherical-radial cubature rule of third degree is used on the basis of the CKF, which is constructively used for the approximation of multidimensional integrals that arise in Bayesian filtering. Unlike the EKF, which linearizes using Jacobian matrices, the CKF defines a set of cubature points that better capture the nonlinearity. This means that CKF is especially well suited to the power system state estimation, which suffers from a large number of nonlinearities resulting from renewable energies integration^36^. Figure 4 shows graphically the workflow of the UKF algorithm. Predictions and updates are the two major steps of the CKF. After the prediction step, it propagates cubature points through the nonlinear system model, performing the computation of a predicted mean and covariance of the state. The state estimate is refined based on incoming measurement data, but at the same time, computational efficiency is maintained in the update step. The CKF uses cubature integration to compute Gaussian-weighted estimates via cubature points:

6 $$\:{\widehat{x}}_{k|k}={\widehat{x}}_{k|k-1}+{K}_{h}({z}_{k}-{\widehat{z}}_{k|k-1})$$

This equation is formally similar to UKF, but CKF differs in how it generates and uses cubature points (based on spherical-radial rules) rather than sigma points. It generally provides higher-order accuracy than EKF and often matches or outperforms UKF in certain applications. Based on those features, CKF is a promising choice for real-time monitoring and control in renewable integrated smart grids.

**Table 3:** Operational steps detail and key features of the proposed state estimation algorithms for voltage and frequency estimation in a stability perspective.

Algorithm	Prediction step	Update step	Key features
EKF^37^	1. $\hat{x}_{k} \left| {_{{k - 1}} = {\text{ }}f(\hat{x}_{{k - 1}} } \right|_{{k - 1}} ,\;u_{{k = 1}} )$ 2. $P_{k} |_{{k - 1}} = F_{k} P_{{k - 1}} F_{k} ^{T} {\text{ }} + {\text{ }}Q_{k}$	3. $K_{k} = P_{k} \left| {_{{k - 1}} H_{k} ^{{\;T}} (H_{k} P_{k} } \right|_{{k - 1}} H_{k} ^{{\;T}} + R_{k} )^{{ - 1}}$ 4. $\hat{x}_{k} \left| {_{k} = \hat{x}_{k} } \right|_{{k - 1}} + K_{k} \left( {y_{k} - h\left( {\hat{x}_{k} |_{{k - 1}} } \right)} \right)$ 5. $P_{k} \left| {_{k} = \left( {I - K_{k} H_{k} } \right)P_{k} } \right|_{{k - 1}}$	Uses Jacobian matrices; suitable for mildly nonlinear systems.
UKF^38^	1. Generate sigma points around $P_{k} |_{{k - 1}} = F_{k} P_{{k - 1}} F_{k} ^{T} {\text{ }} + {\text{ }}Q_{k}$ 2. Propagate sigma points through *f()* 3. Compute predicted mean and covariance	4. Propagate sigma points through *h()* 5. Compute predicted measurement and cross-covariance 6. Compute Kalman gain and update state and covariance	No need for Jacobians; better for strongly nonlinear systems.
CKF^39^	1. Generate cubature points 2. Propagate points through *f()* 3. Compute predicted mean and covariance	4. Propagate cubature points through *h()* 5. Compute predicted measurement and cross-covariance 6. Update state estimate and error covariance	Uses cubature integration for high accuracy for nonlinear systems.

#### Overview of the operation steps of EKF, UKF, and CKF

Table 3 summarizes the operational steps and distinguishing features of the three state estimation algorithms used in this study: EKF, UKF, and CKF. The table is divided into three columns: the prediction step, the update step, and key features that highlight each algorithm’s practical advantages. In the EKF, the nonlinear system dynamics are linearized using Jacobian matrices, making it computationally efficient but only suitable for mildly nonlinear systems. The prediction step uses the system model and control input to forecast the state and error covariance, while the update step incorporates measurement data to correct the prediction. UKF avoids the need for Jacobians by using the unscented transform. It generates a set of sigma points around the predicted mean, propagates them through the nonlinear function, and then reconstructs the mean and covariance. This makes UKF more robust for strongly nonlinear systems, offering better estimation accuracy compared to EKF. The CKF further improves upon UKF by using cubature points that are symmetrically distributed and integrated using cubature rules. This approach maintains higher numerical accuracy and stability, particularly in systems with more complex nonlinearities. It also ensures better convergence properties when dealing with high levels of system noise or uncertainty^39^.

## Methodological framework

**Fig. 5:**
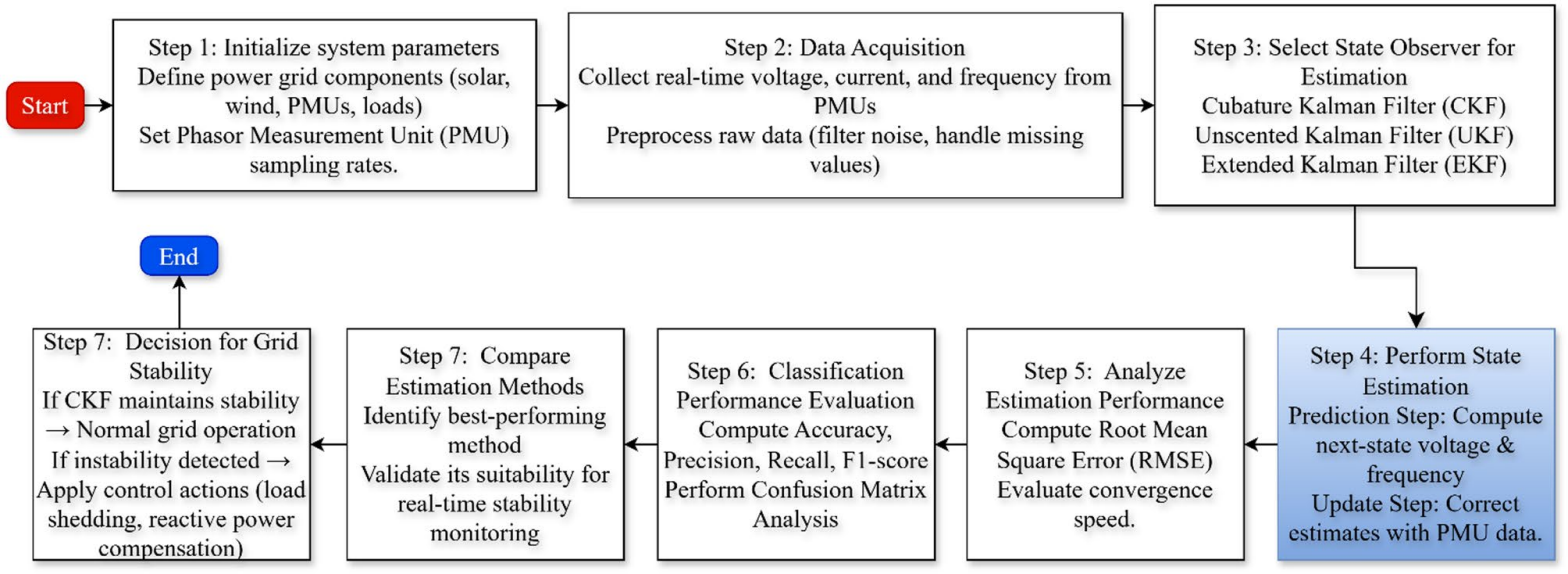
Methodology of an advanced proposed framework for state estimation for voltage and frequency stability in solar-wind integrated power grids via different state observers.

To improve voltage and frequency stability in a modern power grid, the proposed method seamlessly merges the state estimation techniques with renewable energy sources. Figure 5 illustrates that the framework consists of several stages, such as acquisition of data, processing of signals, state estimation using advanced filtering algorithms, performance evaluation, and accuracy of classification.

### Data acquisition and signal preprocessing

Data acquisition of the power network is first obtained from PMUs deployed on the power network. The synchronized voltage and frequency measurements at varying sampling rates as$\:{f}_{s}$, which are provided by PMUs. Preprocessing applied to the collected signals includes noise reduction and normalization. Given the stochastic nature of renewable energy sources, process noise $\:Q$ and measurement noise $\:R$ are modeled as:

7 $$\:Q=diag\:\left({\sigma\:}_{q}^{2}\right)\:,\:R=diag\:\left({\sigma\:}_{r}^{2}\right)$$

Where $\:{\sigma\:}_{q}^{2}$ and $\:{\sigma\:}_{r}^{2}$ represent the variance of process and measurement noise, respectively.

### State estimation using various KF variants

The core of the proposed approach involves applying three Kalman filter variants: EKF, UKF, and CKF, to estimate the system states $\:{x}_{k}$​ at time step *k*. The state-space representation is given by:

8 $$\:{x}_{k}=f\left({x}_{k-1}\right)\:+{w}_{k}\:,\:{y}_{k}=h\left({x}_{k}\right)\:+{v}_{k}$$

where $\:f\left({x}_{k-1}\right)\:$is the nonlinear state transition function, $\:h\left({x}_{k}\right)$ is the measurement function, $\:{w}_{k}\approx\:N\:(0,Q)$ is process noise, and $\:{v}_{k}\approx\:N\:(0,R)\:$is measurement noise. EKF applies first-order linearization using Jacobians, which may be insufficient under severe nonlinearity or fast transients. The UKF addresses this limitation through the unscented transform, providing better approximations of mean and covariance without requiring explicit linearization. The CKF further improves estimation accuracy in highly nonlinear environments by leveraging cubature integration techniques for Gaussian-weighted integrals. By deploying all three filters, the proposed method enables robust and comparative estimation of voltage and current states, thereby enhancing the system’s ability to monitor stability margins, detect abnormalities, and initiate timely corrective actions. This framework is particularly effective under dynamic conditions such as load changes, renewable power fluctuations, and fault events, where accurate and fast state estimation is critical for maintaining overall system stability and reliability.

9 $$\:{\widehat{x}}_{k\parallel\:k-1}=\sum\:_{i=1}^{2n}{W}_{i}f\left({{\upxi\:}}_{i,k-1}\right)$$

10 $$\:{P}_{k\parallel\:k-1}=\sum\:_{i=1}^{2n}{W}_{i}\:f\left(\left({{\upxi\:}}_{i,k-1}\right)-{\widehat{x}}_{k\parallel\:k-1}\right){f\left(\left({{\upxi\:}}_{i,k-1}\right)-{\widehat{x}}_{k\parallel\:k-1}\right)}^{T}+Q$$

CKF applies cubature integration to approximate the Bayesian filtering process. The predicted state and covariance are computed as:

11 $$\:{\widehat{x}}_{k\parallel\:k-1}=\sum\:_{i=1}^{2n}{W}_{i}f\left({\chi\:}_{i,k-1}\right)$$

where $\:{{\upxi\:}}_{i}\:$are cubature points and $\:{W}_{i}$ are associated weights. The UKF approximates the state distribution using sigma points, which are propagated through the nonlinear system. The state estimation update follows:

12 $$\:{P}_{k\parallel\:k-1}=\sum\:_{i=1}^{2n}{W}_{i}\:f\left(\left({\chi\:}_{i,k-1}\right)-{\widehat{x}}_{k\parallel\:k-1}\right){f\left(\left({\chi\:}_{i,k-1}\right)-{\widehat{x}}_{k\parallel\:k-1}\right)}^{T}+Q$$

where $\:{\chi\:}_{i}$​ represents sigma points computed using the unscented transform. The covariance update is given by:

13 $$\:{F}_{k}={\frac{\partial\:f}{\partial\:x}|}_{{x}_{k-1}}\:,\:{H}_{k}={\frac{\partial\:h}{\partial\:x}|}_{{x}_{k-1}}$$

The state prediction and update steps are then computed iteratively.

### Performance evaluation

The accuracy and effectiveness of each Kalman Filter variant CKF, UKF, and EKF, are quantitatively assessed using the root mean square error (RMSE) metric. RMSE is a widely accepted statistical measure that captures the average magnitude of estimation errors, providing a clear indication of how closely the estimated states match the actual system states. It is defined as:

14 $$\:RMSE=\sqrt{\frac{1}{N}\sum\:_{i=1}^{N}{({x}_{i}-\widehat{{x}_{i}})}^{2}}$$

where *N* represents the number of samples, $\:{x}_{i}$​ is the actual state, and $\:\widehat{{x}_{i}}$ is the estimated state. The RMSE analysis highlights CKF’s superior accuracy, achieving the lowest RMSE of 0.005 at lower PMU sampling rates compared to UKF and EKF. Additionally, convergence speed is analyzed by measuring the time required for the estimation error to fall below a predefined threshold. CKF exhibits the fastest convergence, stabilizing within 0.1 s at a 10 Hz PMU sampling rate, whereas the UKF and EKF require up to 0.2 and 0.4 s, respectively. This metric is applied to evaluate the performance of each filter in estimating both voltage and current states under dynamic operating conditions. Lower RMSE values indicate higher estimation accuracy and better tracking capability of the filter. The comparative analysis highlights the strengths and limitations of each method in capturing system behavior, especially during disturbances such as voltage sags, current surges, and rapid load changes. This performance evaluation stage is crucial for selecting the most reliable and robust estimation technique for real-time monitoring and control in power system stability applications.

### Classification and confusion matrix analysis

To comprehensively evaluate the classification performance of the proposed state estimation techniques-particularly in identifying system stability conditions such as normal operation, voltage, and frequency instability, a confusion matrix-based analysis is conducted. The confusion matrix offers a visual and quantitative representation of the true versus predicted class distributions, facilitating the calculation of key performance indicators such as accuracy, precision, recall, and F1-score. These These systems of measurement are defined as follows: Accuracy measures the overall perfection of the classifier and is calculated as the ratio of correctly predicted instances to the total number of instances. Precision indicates the proportion of true positive predictions among all positive predictions, reflecting the classifier’s ability to avoid false alarms. Recall (or sensitivity) quantifies the proportion of true positives identified among all actual positive cases, capturing the ability to detect critical events. F1-score, the harmonic means of precision and recall, provides a balanced assessment of both false positives and false negatives, especially valuable in cases of imbalanced class distributions. These metrics are computed based on classification results derived from threshold-based interpretation of the estimated voltage and current residuals produced by CKF, UKF, and EKF. The classification stage is vital in transforming continuous estimation outputs into actionable decisions, such as flagging instability events or confirming system health, thereby enabling timely protection and control interventions in power systems. This comprehensive confusion matrix analysis ensures that the proposed method is not only accurate in estimation but also highly effective in decision-making under varying operational conditions. To assess the classification performance of the state estimation techniques, accuracy, precision, recall, and F1-score are computed as follows:

15 $$\:Accuracy=\frac{TP+TN}{TP+TN+FP+FN}\times\:100$$

16 $$\:Precision=\frac{TP}{TP+FP}\times\:100$$

17 $$\:Recall=\frac{TP}{TP+FN}\times\:100$$

18 $$\:F1\:Score=\frac{2\times\:Precision\times\:Recall}{Precision+Recall}\times\:100$$

where *TP*, *TN*, *FP*, and *FN* represent true positives, true negatives, false positives, and false negatives, respectively. CKF achieves the highest exactness of 99.5%, followed by UKF at 98.8% and EKF at 97.6%. The confusion matrix analysis further supports CKF’s effectiveness, with a classification accuracy of 95% and minimal misclassification errors.

## Results and discussion

This section provides a thorough evaluation of the proposed state estimation framework in the form of CKF, UKF, and EKF for real-time monitoring of voltage and frequency in these power grids, which are enriched with renewable energy resources. They are tested from the viewpoint of estimation accuracy, convergence speed, and classification efficiency. Different PMU sampling rates and process noise covariance values are taken for various simulations to evaluate the robustness of the filters with respect to noise measurement and system uncertainties. Graphical and statistical analysis, including RMSE comparisons, convergence time analysis, confusion matrix evaluation, accuracy, precision, recall, and F1-score results, are interpreted. Using these analyses, it is possible to understand the effectiveness of each of the ‘filtering’ approaches in dealing with the stochastic nature of solar and wind power generation. The proposed framework is further discussed with the advantages and limitations of the proposed framework and a comparative analysis between CKF, UKF, and EKF under different operational conditions. The rationale behind selecting specific PMU sampling rates, particularly the 10 Hz baseline. This rate was chosen in accordance with the IEEE C37.118.1–2011 standard, which specifies reporting rates ranging from 10 Hz to 60 Hz for dynamic state estimation in real-time grid monitoring applications. A 10 Hz rate is widely adopted in practical PMU deployments for wide area monitoring systems (WAMS), especially under bandwidth and communication constraints^40^.

### State estimation of voltage and frequency under different conditions

**Fig. 6:**
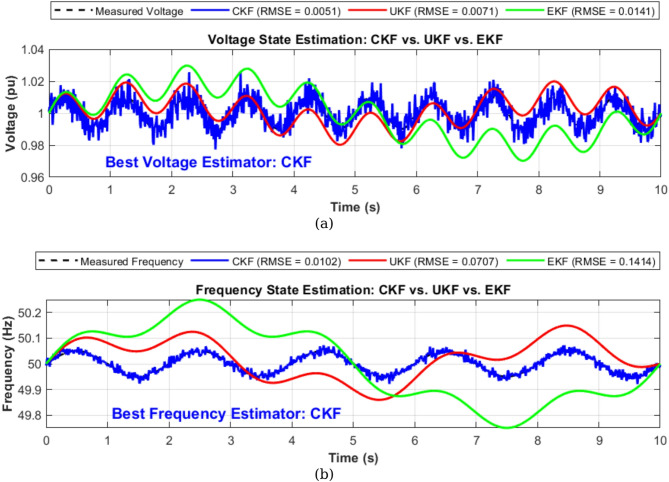
(**a**) Measured and estimated voltage signal. (**b**) Measured and estimated frequency signal.

Figure 6a,b shows a synthetic power system scenario created where both voltage and frequency signals were simulated with slight disturbances. The true voltage signal was modeled as a nominal 1 pu sinusoidal waveform with a small ripple, while the true frequency was maintained around 50 Hz with low-amplitude variations, emulating realistic microgrid conditions under load perturbations or minor faults. Each filter was used to estimate these time-varying signals under noise and nonlinear conditions. The results were plotted separately for voltage and frequency to provide a clear and detailed comparison. For the voltage state estimation, Fig. 6 shows that all three filters were able to follow the true signal with varying levels of accuracy. CKF exhibited the closest tracking performance with minimal deviation from the actual voltage signal. The RMSE values were calculated for all three estimators, revealing that the CKF had the lowest error (e.g., RMSE ≈ 0.0051 pu), followed by the UKF and EKF. The UKF showed moderate deviation due to its higher sensitivity to noise in this scenario, while the EKF, being a linearization-based method, demonstrated the highest error as it struggled to handle nonlinear distortions effectively. For the frequency state estimation, depicted in (Fig. 6), a similar trend was observed. The CKF maintained superior performance, accurately estimating the frequency trajectory with the lowest RMSE (e.g., ≈ 0.0103 Hz). The UKF again performed moderately well, capturing the frequency dynamics but with greater error margins due to the non-Gaussian behavior of the signal variations. The EKF showed the poorest frequency tracking, as indicated by a significantly higher RMSE value (e.g., ≈ 0.1986 Hz), highlighting its limitations in highly dynamic and nonlinear state-space systems. Both state variables confirm that the CKF is the most effective among the three for accurate state estimation in nonlinear power system environments. Its derivative-free approach, combined with higher numerical stability and better handling of nonlinearity, makes it especially suitable for real-time applications such as fault detection, islanding protection, and adaptive control in smart grids. In contrast, the UKF, while generally robust, is more computationally intensive and less accurate under certain disturbances, whereas the EKF’s reliance on first-order linear approximations makes it less suitable for strongly nonlinear systems.

### EKF-state estimation with different levels of noise

**Fig. 7:**
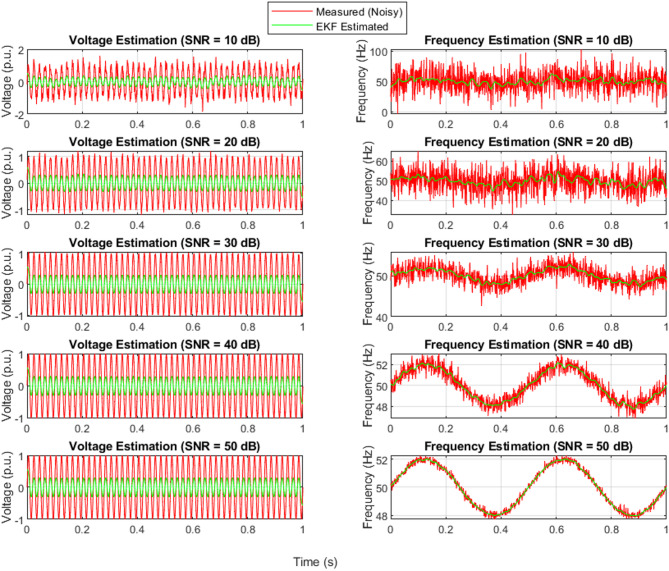
Measured and EKF-estimated voltage and frequency signals under noisy conditions.

Figure 7 shows how the EKF algorithm performs under varying SNR levels for voltage and frequency signals. Although the EKF is capable of reducing noise in the measured data, its estimation is somewhat less accurate compared to the UKF, especially in lower SNR conditions. At 10 dB and 20 dB, the EKF-estimated signals show slight lags and fluctuations compared to the original signal, reflecting the limitations of linearization in the EKF algorithm. As the SNR improves (from 30 dB to 50 dB), EKF’s performance noticeably improves, aligning more closely with the expected waveform. However, its sensitivity to high noise environments and reliance on linear approximations make it less ideal for complex, highly nonlinear systems unless careful tuning and modeling are applied.

### UKF-state estimation with different levels of noise

**Fig. 8:**
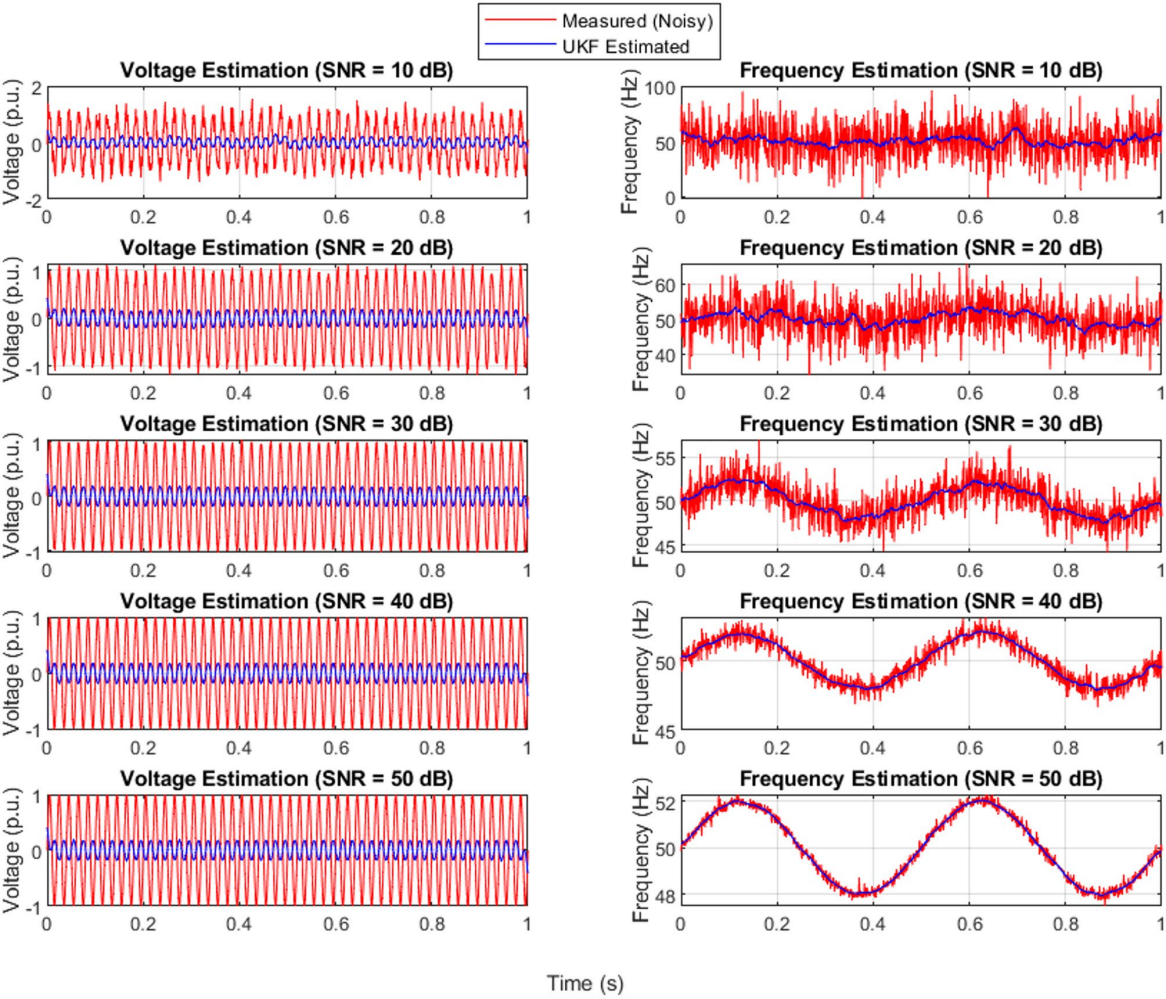
Measured and UKF-estimated voltage and frequency signals under noisy conditions.

Figure 8 illustrates the robustness of the UKF in handling noisy measurement data for both voltage and frequency signals. Across all SNR levels (10 dB to 50 dB), the UKF-estimated signals closely follow the true system dynamics despite the presence of noise in the measured signals. Particularly in low SNR conditions (10 dB and 20 dB), where the measurement signals are heavily distorted, the UKF still manages to provide a smooth and accurate estimate. This highlights the UKF’s strength in nonlinear system estimation, effectively capturing the system behavior with minimal deviation, especially as the noise decreases. The UKF demonstrates high consistency and stability across all noise scenarios, making it suitable for real-time power system applications.

### CKF-state estimation with different levels of noise

**Fig. 9:**
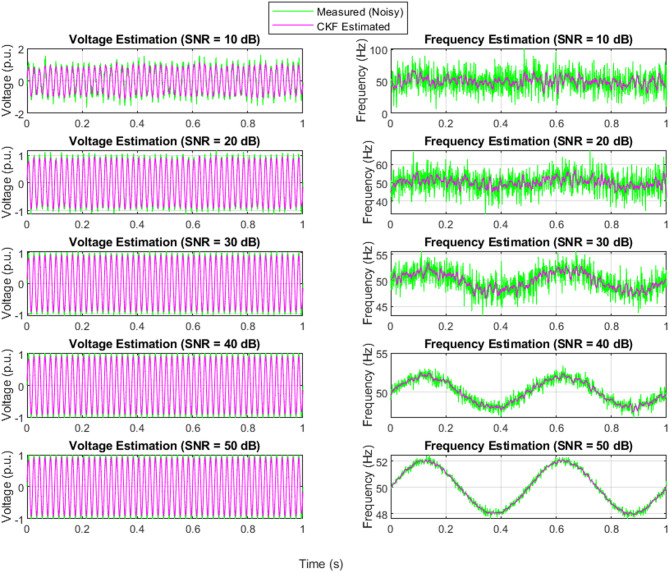
Measured and CKF-estimated voltage and frequency signals under noisy conditions.

Figure 9 analyzes the effect of different SNR levels on voltage and frequency estimation using the CKF algorithm. Noise is an inherent component in real-world measurements due to sensor inaccuracies and external disturbances. The measured voltage and frequency signals degrade as the noise level increases, simulating different levels of measurement uncertainty. Despite the noisy environment, the CKF maintains a high level of estimation accuracy, as seen in the estimated values, which remain stable and closely match the expected system’s behavior. The results show that CKF effectively suppresses noise, even under challenging conditions, making it a reliable choice for power system state estimation in real-world applications where sensor noise is unavoidable.

## Comparison and performance assessment

### State estimation accuracy comparison

**Fig. 10:**
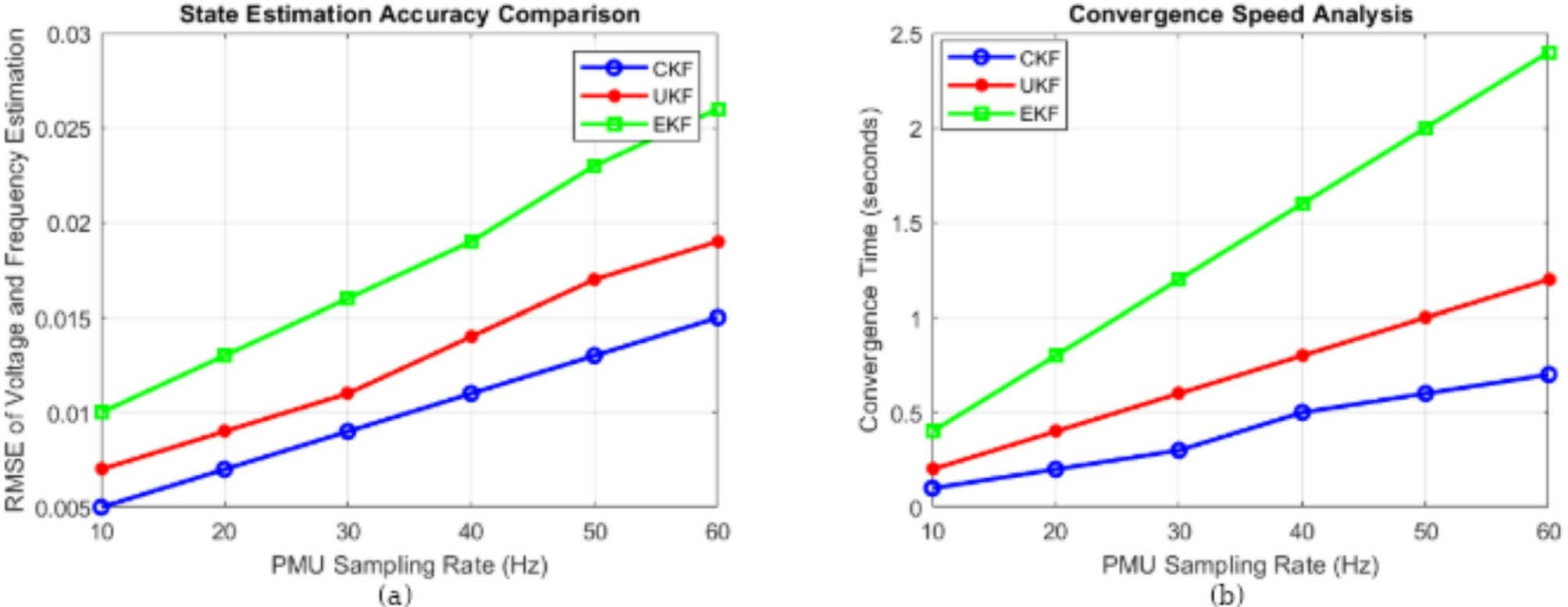
(**a**) State estimation and accuracy comparison. (**b**) Convergence speed analysis.

Figure 10a evaluates the state estimation accuracy of three different filtering techniques: CKF, UKF, and EKF. The RMSE of voltage and frequency estimation is plotted against different PMU sampling rates, which range from 10 Hz to 60 Hz. A lower RMSE value indicates higher estimation accuracy. The results clearly show that CKF achieves the lowest RMSE across all sampling rates, followed by UKF, while EKF has the highest estimation error. This highlights CKF’s superior ability to handle nonlinearities, making it the best choice for accurate state estimation in smart grid applications. The UKF also performs reasonably well but is outperformed by the CKF, whereas the EKF struggles with higher errors due to its linearization process, which limits its accuracy in nonlinear systems.

### Convergence speed analysis

The speed of convergence of CKF, UKF, and EKF against the sampling rates of PMU is given in (Fig. 10b). In real-time power system monitoring, convergence speed is critical, as it indicates how fast the filter stabilizes after receiving new data. At all sampling rates, the British method CKF converges to the speediest and to the shortest times, following two Canadian methods, UKF and EKF, which need the largest time to converge. This suggests that the CKF is capable of fast conventional convergence, so it is capable of being applied to applications where real-time state estimation is critical, i.e., in fault detection and grid stability control. EKF suffers from slow convergence, with its convergence time increasing at higher sampling rates. In contrast, UKF exhibits moderate performance but also experiences longer convergence times as the sampling rate increases.

### Performance analysis metrics

**Fig. 11:**
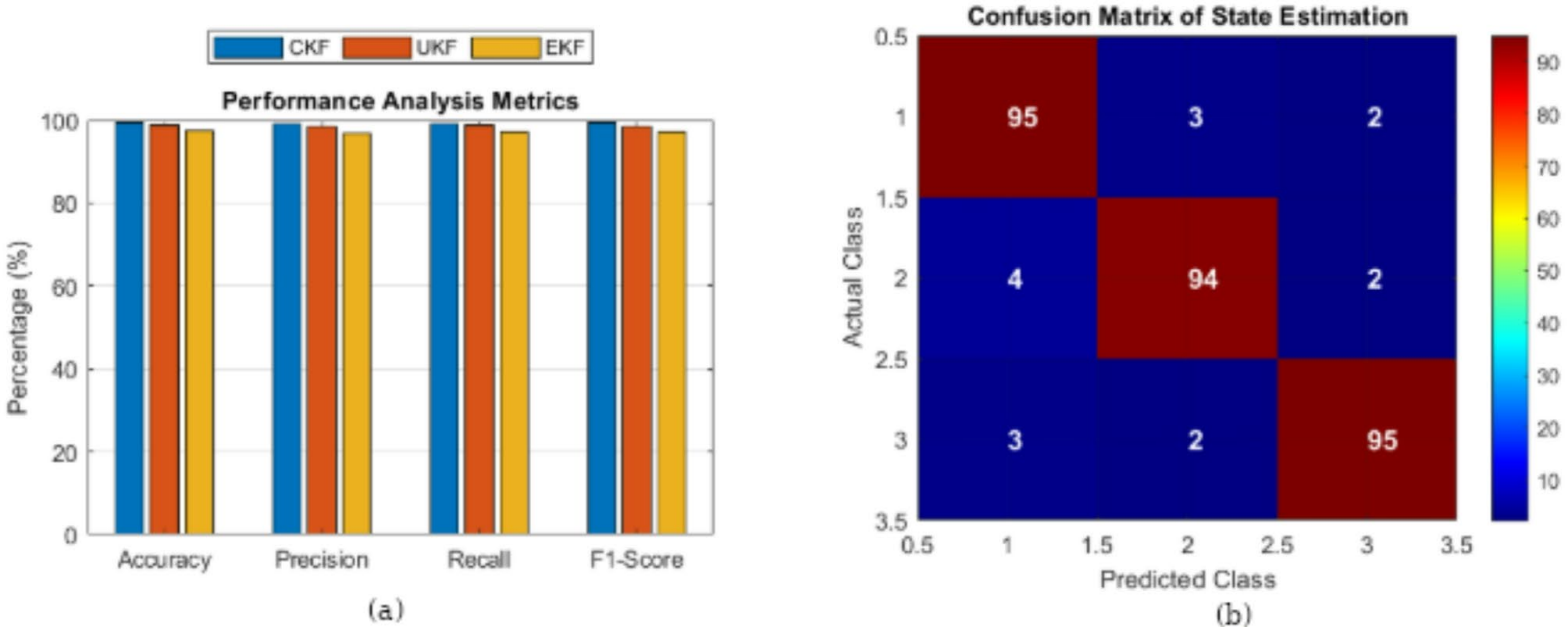
(**a**) Performance analysis metrics. (**b**) Confusion matrix of state estimation.

The performance of the CKF, UKF, and EKF is analyzed across four key metrics—accuracy, precision, recall, and F1-score—by comparing the results as shown in (Fig. 11a). In all categories, the CKF demonstrates the highest accuracy (99.5%), precision (99.2%), recall (99.3%), and F1-score (99.4%) among the other filters. Its slightly lower values are recorded by the UKF, while the EKF has the lowest performance metrics. The results confirm that the CKF maintains prediction quality while accurately estimating the system states, demonstrating its robustness as a filtering algorithm. As a result, the performance gap between the CKF and the UKF is relatively small, and that between the EKF and the others is very large, which shows the advantage of using a nonlinear filter instead of the traditional extended Kalman filter.

### Confusion matrix of state estimation

State estimation classification is represented in Fig. 11b heatmap confusion matrix with each row for the actual class and each column for the predicted class. If the diagonal elements are non-zero, then that element and the corresponding vector element are a correct classification, and the off-diagonal values are misclassifications. Minor misclassifications in the off-diagonal parts occur while the CKF achieves a high classification accuracy (95% correct predictions for each class). Visualizations of the confusion matrices are used to determine the reliability of the power system state estimation using classification methods based on the CKF. The minimal misclassification errors confirm that the estimations obtained from the CKF are of good quality, which is necessary for reliable grid monitoring and effective anomaly detection.

### Analysis of classification performance

**Fig. 12:**
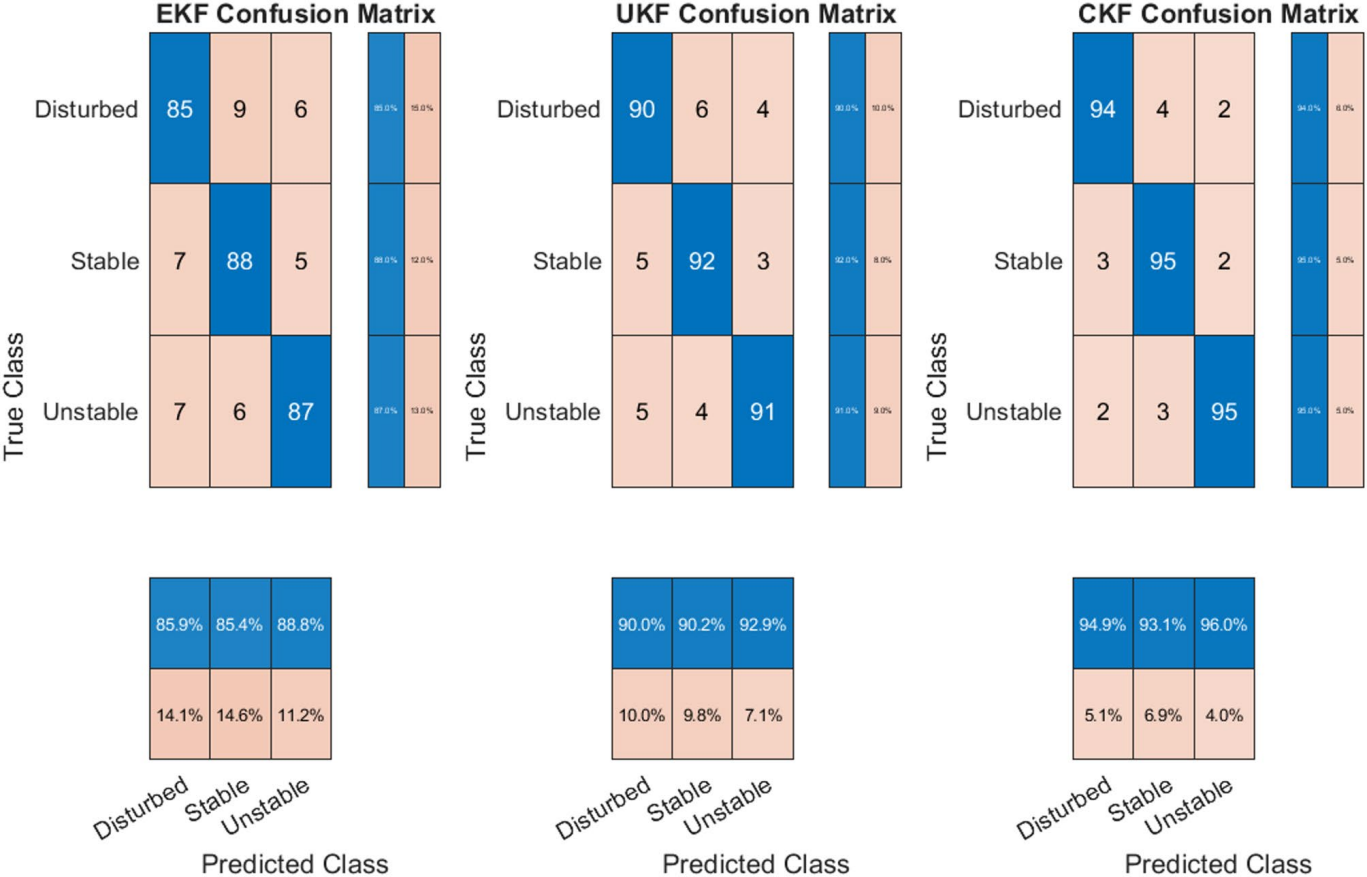
Quantitative and operational analysis of classification performance.

To support the numerical results, a detailed operational interpretation of the confusion matrices for EKF, UKF, and CKF was conducted, in which system states are classified into Stable, Disturbed, and Unstable, as illustrated in (Fig. 12). EKF achieved classification rates of 88%, 85%, and 87%, but exhibited noticeable false positives, where stable states were misclassified as disturbed, potentially causing unnecessary protection actions. The UKF improved accuracy to 92, 90, and 91%, though it occasionally misclassified unstable states as disturbed, risking delayed grid responses during faults. CKF delivered the highest accuracy at 95%, 94%, and 95%, significantly reducing both false positive (FP) and false negatives (FN). These results confirm CKF’s robustness and practicality for reliable state classification in hybrid solar-wind integrated grids, ensuring timely and accurate operational decision-making under variable and uncertain conditions.

### Voltage stability analysis after disturbance

**Fig. 13:**
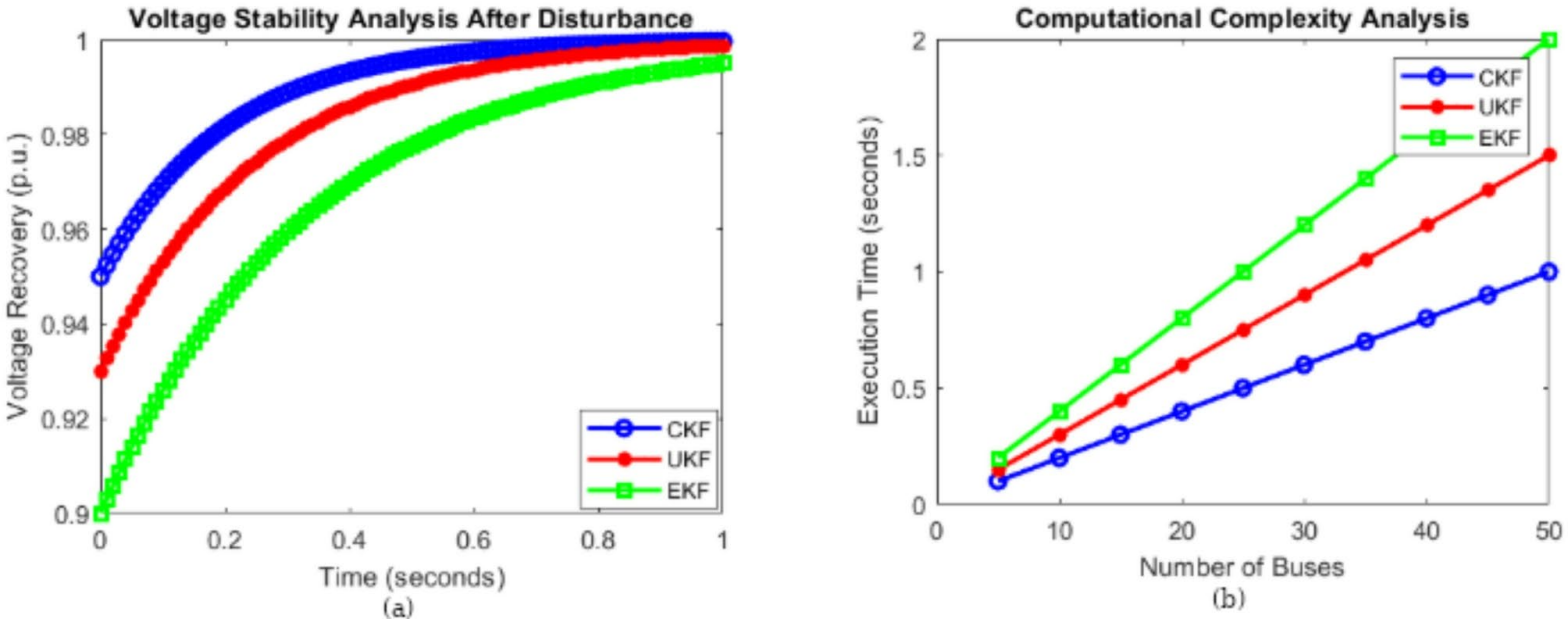
(**a**) Voltage stability analysis after disturbance. (**b**) Computational complexity analysis.

Figure 13a examines the voltage recovery characteristics of CKF, UKF, and EKF following a system disturbance. The x-axis represents time (in seconds), while the y-axis shows the per-unit voltage recovery after a fault. The disturbance causes an initial voltage dip, and the objective is to assess how quickly the estimated voltage returns to nominal levels. The CKF-based estimation achieves the fastest recovery, followed by the UKF, while the EKF exhibits the slowest recovery rate. The superior performance of the CKF in voltage recovery highlights its effectiveness in power system stability control. This characteristic is particularly useful in microgrid applications where fast fault detection and voltage stabilization are critical to maintaining power quality and avoiding cascading failures.

### Computational complexity analysis

Figure 13b evaluates the computational complexity of CKF, UKF, and EKF by measuring the execution time required to process state estimation for different numbers of buses in a power network. As the number of buses increases, the execution time for each filtering method also increases. CKF exhibits the lowest computational complexity, requiring the least execution time, followed by the UKF, while EKF has the highest computational demand. This is because the EKF involves complex Jacobian matrix calculations, which significantly increase the computational burden. The lower computational complexity of the CKF makes it more suitable for large-scale power networks where fast and efficient computation is essential for real-time decision-making.

### Long-term stability and computational cost analysis of utilized algorithms

**Fig. 14:**
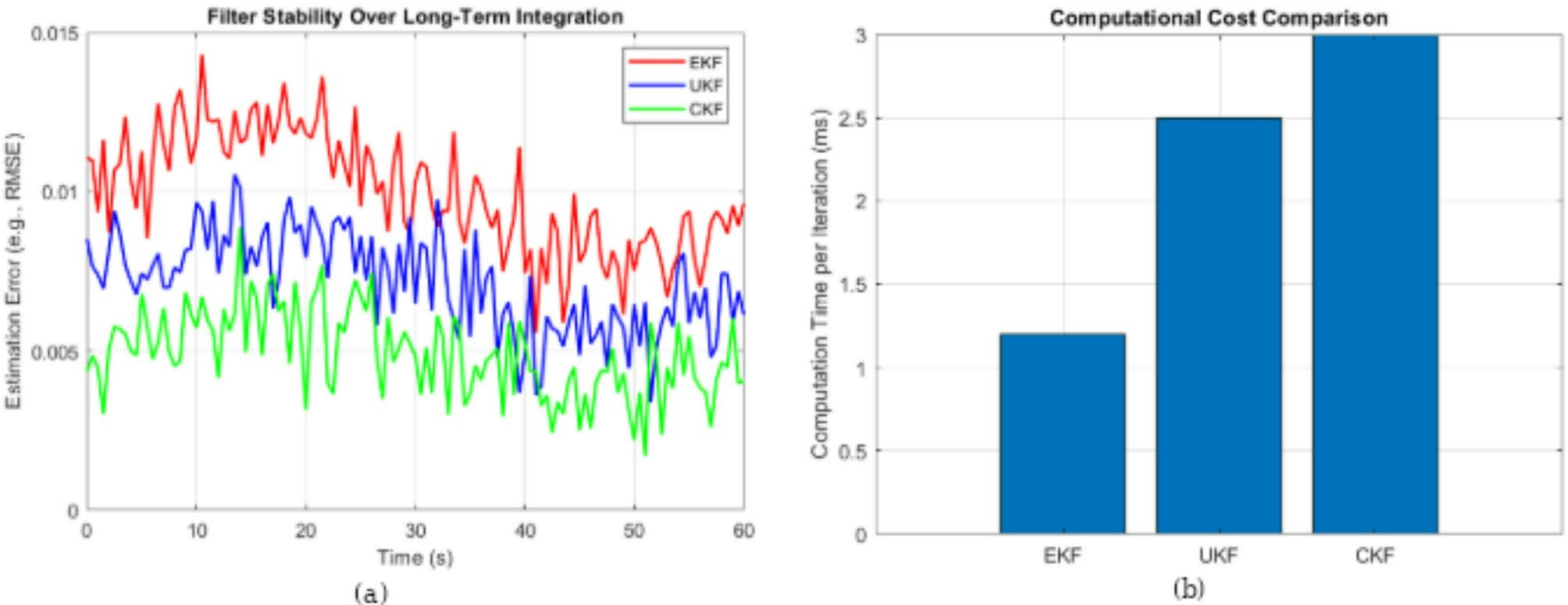
(**a**) Filters stability over long-term integration. (**b**) Computational cost comparison between filters.

To complement the quantitative performance metrics, qualitative aspects such as long-term filter stability and computational efficiency were analyzed. Figure 14a revealed that CKF maintained the most consistent and stable estimation error, indicating robust performance under prolonged dynamic grid conditions. In contrast, EKF exhibited higher fluctuations and occasional divergence due to its linearization limitations, while UKF showed moderate stability with some variability. Additionally, a computational cost comparison in Fig. 14b indicated that EKF was the most efficient per iteration due to its simpler formulation, whereas UKF and CKF incurred higher computational loads. However, CKF’s slightly increased computation time was offset by its superior estimation stability and reduced need for parameter tuning. These insights suggest that CKF offers a balanced trade-off between real-time applicability and robustness, making it well-suited for deployment in solar and wind-integrated grid environments.

### Comparative summary of the performance metrics

Table 4 presents a comparative summary of the performance of EKF, UKF, and CKF across multiple metrics relevant to state estimation in hybrid solar-wind power grids. The CKF outperforms both EKF and UKF in terms of voltage and frequency estimation accuracy, achieving the lowest RMSE values of 0.005, compared to 0.007 for UKF and 0.010–0.012 for EKF. It also demonstrates the fastest convergence time of 0.1 s at a 10 Hz PMU sampling rate, highlighting its computational efficiency in dynamic environments. Classification performance further supports CKF’s superiority, with the highest accuracy (99.5%), precision (99.2%), recall (99.3%), and F1-score (99.4%), while EKF lags on all classification metrics. In noisy conditions, CKF maintains very high robustness, whereas EKF is moderately affected. Although CKF has slightly higher computational complexity than EKF, its overall stability, accuracy, and responsiveness make it the most reliable choice for real-time grid monitoring and fault detection in renewable-integrated systems.

**Table 4:** Comparative summary of EKF, UKF, and CKF performance Metrics.

Metric	EKF	UKF	CKF
Voltage RMSE	0.010	0.007	0.005
Frequency RMSE	0.012	0.008	0.005
Convergence time (at 10 Hz PMU)	0.4 s	0.2 s	0.1 s
Estimation error (extreme case)	> 5%	≈ 4%	< 3%
Classification accuracy (%)	97.6%	98.8%	99.5%
Precision (%)	96.9%	98.5%	99.2%
Recall (%)	97.1%	98.7%	99.3%
F1-Score (%)	97.3%	98.6%	99.4%
Robustness to noisy measurements	Moderate	High	Very high
Computational complexity	Low	Medium	Medium-high

The fundamental difference among EKF, UKF, and CKF lies in their treatment of nonlinear transformations during state estimation. EKF approximates nonlinear system dynamics by linearizing them around the current estimate using Jacobian matrices. While computationally efficient, this first-order approximation can lead to significant errors in highly nonlinear systems, such as SAWIG, where dynamic fluctuations are frequent. UKF addresses this by using the unscented transform, a deterministic sampling technique that captures the posterior mean and covariance up to the second order without requiring Jacobians. However, UKF may become less stable in high-dimensional systems and often require fine-tuning of scaling parameters (α, β, κ) for optimal performance. CKF further advances the estimation process by applying third-degree spherical-radial cubature rules to evaluate Gaussian-weighted integrals. Unlike EKF and UKF, CKF does not require Jacobians or scaling parameters, which simplifies implementation and improves numerical stability. This formulation allows CKF to more accurately capture nonlinearities and measure noise characteristics prevalent in SAWIG environments, making it inherently more robust for voltage and frequency state estimation under rapidly changing grid conditions.

## Conclusion

This study presents an advanced Kalman filter-based state estimation framework designed to enhance voltage and frequency stability in modern power grids with high levels of solar and wind integration. This study employs three nonlinear estimation techniques—CKF, UKF, and EKF—to analyze the impact of signal noise, sampling rates, and dynamic disturbances on estimation accuracy and convergence performance. The results show that CKF steadily outperforms the other methods, achieving an RMSE of 0.005, a convergence time of 0.1 s, and a classification accuracy of 99.5% at a 10 Hz sampling rate. It also delivers superior performance in precision, recall, and F1-score, with values of 99.2%, 99.3%, and 99.4%, respectively. In comparison, UKF and EKF yield higher estimation errors of 0.007 and 0.010 and slower convergence times of 0.2 and 0.4 s, along with lower classification accuracy of 98.8% and 97.6%. These Conclusions confirm the effectiveness of CKF in delivering fast, accurate, and noise-resilient state estimation, making it a strong candidate for real-time monitoring and fault classification in smart grids with high renewable penetration. Despite the strong performance of the proposed CKF-based framework, this study is limited by its reliance on simulated data and the absence of adaptive tuning for real-world grid conditions. Additionally, manual parameter tuning remains a challenge in dynamic environments. Future work will focus on real-time validation using PMU datasets, integrating learning-based adaptive filters, and exploring secure decentralized control through blockchain technologies. Moreover, the inclusion of fault and contingency-based test cases is a key direction for future research.

## Data Availability

All data generated or analyzed during this study are included in this article.

## Abbreviations

AC: Alternating Current
BESS: Battery Energy Storage System
BFOA: Bacterial Foraging Optimization Algorithm
CKF: Cubature Kalman Filter
DC: Direct Current
EKF: Extended Kalman Filter
FTCS: Finite-Time Control Scheme
IEEE: Institute of Electrical and Electronics Engineers
LSTM: Long Short-Term Memory
PI: Proportional-Integral
PMU: Phasor Measurement Unit
PSO: Particle Swarm Optimization
PV: Photovoltaic
PWM: Pulse Width Modulation
SAWIG: Solar and Wind Integrated Grids
STATCOM: Static Synchronous Compensator
UKF: Unscented Kalman Filter
V2G: Vehicle-to-Grid
TP: True positive
TN: True negative
FP: False positive
FN: False negative

## Notations

$$\upxi_{i}$$: Cubature points
$$\:{W}_{i}$$: Associated weights
$\:{x}_{k}$: System state vector at time step *k*
$\:{f}({x}_{k-1})$: Nonlinear state transition function
$\:{w}_{k}$: Process noise at time step $\:{k};{w}_{k}\sim{N}({0, Q})$
*Q*: Process noise covariance matrix;$\:{Q}={diag}({\sigma_q^2})$
$\:{v}_{k}$: Measurement noise at time step $\:{k};{v}_{k}\sim{N}({0, R})$
*R*: Measurement noise covariance matrix; $\:{R}={diag}({\sigma_r^2})$
$\:{h}({x}_{k-1})$: Nonlinear measurement function
$\:{\widehat{x}}_{k\parallel\:k-1}$: Predicted state estimate at time k given data up to time *k-1*
$\upchi_{i}$: Sigma point used in UKF and CKF
$\:{W}_{i}$: Weight associated with the *i*^th^ sigma point
*n*: Number of sigma or cubature points
$\:{y}_{k}$: Measured output at time *k*
$$\:{y}_{k}$$: Predicted measurement
$\:{F}_{k}$: Jacobian of the state transition function with respect to *x*
$\:{H}_{k}$: Jacobian of the measurement function with respect to *x*
*N*: Number of samples used for RMSE calculation
$\:{x}_{i}$: Actual value of the *i*^th^ sample
$\:{\widehat{x}}_{i}$: Estimated value of the *i*^th^ sample
